# Efficiency of Long Lateral Mass Screw in Posterior Cervical Fusion

**DOI:** 10.7759/cureus.65139

**Published:** 2024-07-22

**Authors:** Seiya Watanabe, Kazuo Nakanishi, Kazuya Uchino, Hideaki Iba, Yoshihisa Sugimoto, Shigeru Mitani

**Affiliations:** 1 Orthopaedics, Kawasaki Medical School, Okayama, JPN

**Keywords:** laminectomy, laminoplasty, pedicle screw, lateral mass screw, cervical

## Abstract

Introduction: Long lateral mass screw (LLMS) technique for posterior cervical fusion has been performed in our hospital since 2019. In this study, the LLMS insertion technique, deviation rate, and insertion torque have been described. Moreover, several major concerns associated with LLMS have been adequately addressed.

Methods: This study included 58 patients (43 men and 15 women) who had undergone LLMS surgery at our hospital during the four-year period from December 2019 to December 2023, and were evaluated using postoperative CT. The evaluation parameters included the screw length at each vertebral segment, screw angle in the sagittal section, distance between the screw heads, and complications.

Results: The median screw length at C3 was 23.0 mm (22.0-24.0 mm), the screw angle was 36.1° (31.6-41.8°), and the distance between screw heads was 13.8 mm (11.6-17.2 mm). The median screw length at C4 was 22.0 mm (21.0-24.0 mm), the screw angle was 36.2° (28.7-40.7°), and the distance between screw heads was 15.9 mm (13.0-19.0 mm). The median screw length at C5 was 21.0 mm (20.0-22.0 mm), the screw angle was 35.6° (28.0-39.7°), and the distance between screw heads was 17.6 mm (15.1-20.4 mm). The median screw length for C6 was 20.0 mm (19.0-22.0 mm), the screw angle was 29.2° (25.2-36.8°), and the distance between screw heads was 20.4 mm (16.1-24.4 mm).

Conclusion: The major limitations of the LLMS technique were inadequate screw angle, difficulty inserting long screws, inadequate decompression, and the inability to perform cervical laminoplasty. However, these limitations did not substantially affect the efficiency of LLMS. LLMS has fewer complications and can insert longer screws than LMS.

## Introduction

Surgical stabilization is necessary for patients with cervical spine instability. Posterior cervical fusion with a pedicle screw (PS) or lateral mass screw (LMS) is widely used. However, despite their excellent fixation strength, complications due to deviation during PS insertion remain challenging [1]. LMS is a safe and simple technique; however, fracture of the lateral mass can result in implant dislocation [2].

Herein, we report the long lateral mass screw (LLMS) technique, which is safer than the PS and has a higher fixation force than the LMS. The LLMS insertion method [3], rate of deviation [4], and insertion torque value [5] have been elaborated. We believe LLMS is a simple and useful screw insertion method. However, there are three problems with the LLMS. The first regards the angle of the screw. In cases with thick neck skin, the screw angle is insufficient. Second, the proximity of the screw insertion point prevents adequate decompression. Third, it cannot be combined with cervical laminoplasty. The purpose of this study is to examine whether these LLMS problems are clinical weaknesses.

## Materials and methods

This study included 58 patients (43 men and 15 women) who had undergone posterior cervical fusion using LLMS at the Kawasaki Medical School, Kurashiki City, Okayama Prefecture, Japan, during the four-year period from December 2019 to December 2023 and were evaluated using postoperative computed tomography (CT). The study was approved by the Ethics Review Committee of Kawasaki Medical School (approval number: 3782).

The average age was 68.9 years old. The study did not consider BMI, race, or medical history. In total, 36 fusions and 22 decompression fusions were performed. Cervical spinal cord injury, cervical spine injury, cervical myelopathy, cervical posterior longitudinal ligament ossification, cervical spine dislocation fracture, and metastatic spinal tumors were reported in 23, 16, 9, 6, two, and two patients, respectively.

**Figure 1:**
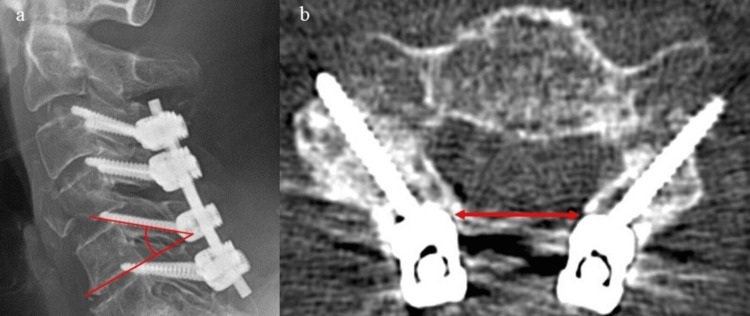
Screw angle and distance between screw heads a: Screw angle (the angle between the extension of the inferior vertebral margin of each vertebra and the screw). If the angles of the left and right screws were different, the average angle was used. b: Distance between screw heads was measured as the medial distance between the left and right screw heads. Postoperative CT axial images were used for evaluation.

The evaluation parameters were the screw length at each vertebral segment, screw angle in the sagittal section, distance between the screw heads, and complications. The angle of the screw was evaluated using postoperative radiography and CT to assess the angle between the extension of the inferior vertebral margin of each vertebra and the screw (Figure 1a). The distance between the screw heads was measured as the medial distance between the left and right screw heads on the axial postoperative CT images (Figure 1b).

Procedure

**Figure 2:**
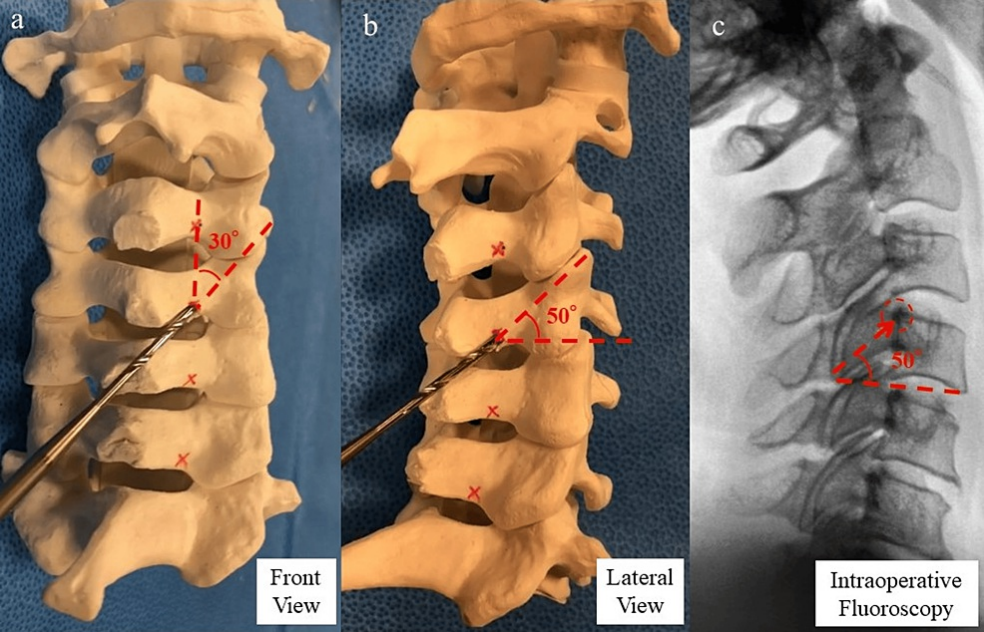
How to insert LLMS a: The insertion point of the LLMS was 2 mm cephalad and 2 mm medial to the medial edge of the facet joint. The insertion angle was 50° cephalad. b: The insertion angle was 30° lateral c: Intraoperative fluoroscopy targets the angle between the superior margin of the vertebral body and the posterior wall of the vertebral body, parallel to the facet joint LLMS: long lateral mass screw

The longest LMS insertion was measured from the third to sixth cervical vertebrae using a ZedSpine (Lexi Co. Ltd, Tokyo, Japan). The insertion point of the LLMS was 2 mm cephalad and 2 mm medial to the medial edge of the facet joint. The insertion angles were 50° cephalad and 30° lateral (Figure 2a, 2b). Intraoperative fluoroscopy targeted the angle between the superior margin of the vertebral body and the posterior wall of the vertebral body, parallel to the facet joint (Figure 2c).

The ideal screw length for the LLMS is 24 mm on average [3,4].

Statistical analysis

The screw length, angle, and distance between screw heads are presented as median values (interquartile range (IQR)). There is a correlation between screw length and fixation force. Therefore, we believe that examining the length and angle of the screw is an examination of the fixation force of the LLMS. Then, by measuring the distance between the screw heads, it is possible to determine whether sufficient decompression can be achieved when performed. The Kruskal-Wallis H-test was also used to statistically evaluate whether there were significant differences in screw angles and the distance of screw heads between each vertebra. A p-value <0.05 was considered statistically significant.

## Results

The median screw length at C3 was 23.0 mm (IQR 22.0-24.0 mm), screw angle was 36.1° (IQR 31.6-41.8°), and the distance between screw heads was 13.8 mm (IQR 11.6-17.2 mm). The median screw length of C4 was 22.0 mm (IQR 21.0-24.0 mm), the screw angle was 36.2° (IQR 28.7-40.7°), and the distance between screw heads was 15.9 mm (IQR 13.0-19.0 mm). The median screw length for C5 was 21.0 mm (IQR 20.0-22.0 mm), the screw angle was 35.6° (IQR 28.0-39.7°), and the distance between screw heads was 17.6 mm (IQR 15.1-20.4 mm). The median screw length for C6 was 20.0 mm (IQR 19.0-22.0 mm), the screw angle was 29.2° (IQR 25.2-36.8°), and the distance between screw heads was 20.4 mm (IQR 16.1-24.4 mm) (Table 1).

**Table 1:** Results of screw length, screw angle, and distance between screw heads Data given as median (IQR) IQR: interquartile range

Evaluation parameter	C3	C4	C5	C6
Screw length, median (IQR)	23.0 mm (22-24mm)	22.0 mm (21-24 mm)	21.0 mm (20-22 mm)	20.0 mm (19-22 mm)
Screw angle, median (IQR)	36.1° (31.6-41.8°)	36.2° (28.7-40.7°)	35.6° (28.0-39.7°)	29.2° (25.2-36.8°)
Distance between screw heads, median (IQR)	13.8 mm (11.6-17.2 mm)	15.9 mm (13.0-19.0 mm)	17.6 mm (15.1-20.4 mm)	20.4 mm (16.1-20.4 mm)

**Figure 3:**
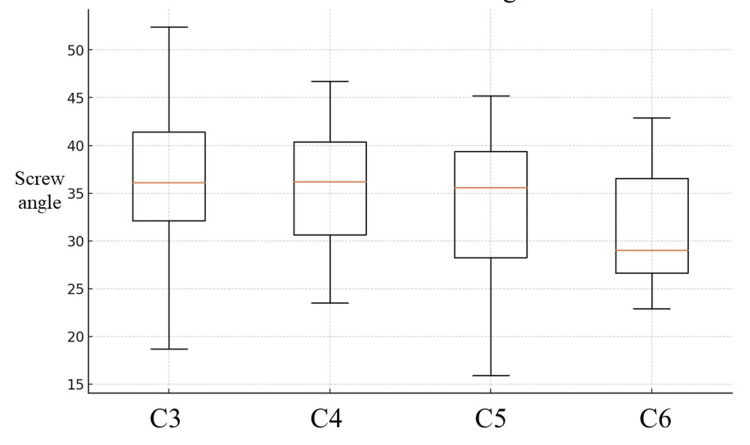
Box plot of screw angle The distribution of screw angles between each vertebra is shown. Each box represents the interquartile range of the data for each group, with the median indicated by the red line. The whiskers above and below the boxes indicate the range of the data.

There was no significant difference among the four groups with screw angle (P = 0.054) (Figure 3).

**Figure 4:**
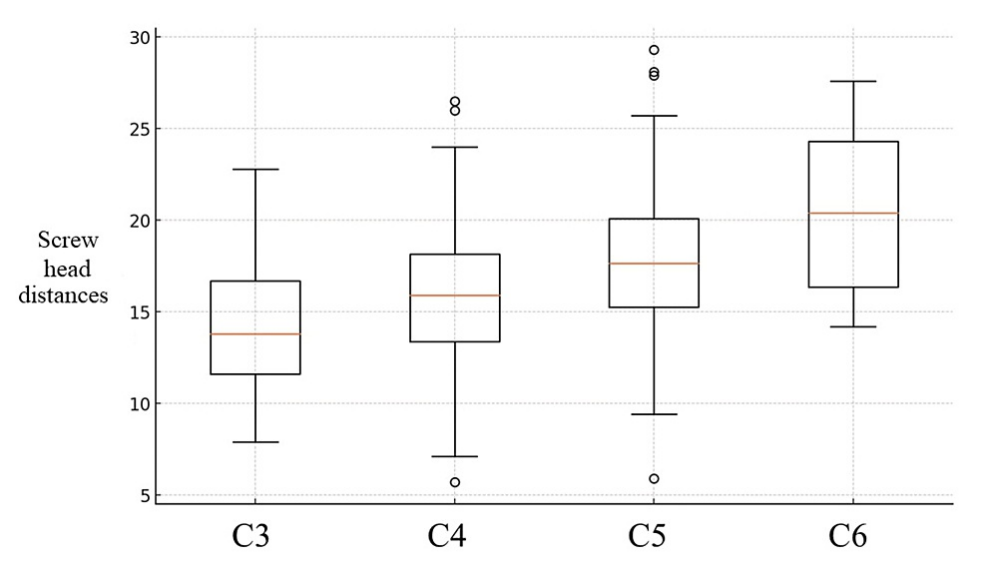
Box plot of screw head distances The distribution of the distance of screw head between each vertebra is shown. Each box represents the interquartile range of the data for each group, with the median indicated by the red line. The whiskers above and below the boxes indicate the range of the data.

There was a significant difference between the four groups with screw head distance (P = 0.001) (Figure 4).

One case of C5 palsy occurred, but this was due to decompression, not screw deviation. There were no neurovascular injuries and kyphotic deformity.

## Discussion

To date, the following three major limitations have been identified with LLMS technique. First, insertion of a long screw is difficult in patients with thick subcutaneous tissue in the posterior neck, and insufficient angling can lead to improper screw placement. Second, the screw insertion points on both sides are in proximity to each other to provide sufficient decompression width. Third, because cervical arthroplasty cannot be used in combination with cervical vertebroplasty, only cervical laminectomy can be used for decompression. The ideal insertion of the LLMS is to angle it 50° cephalad, parallel to the facet joint [3]. The insertion angles for C3-C6 used in this study were 36.1°, 36.2°, 35.6°, and 29.2°, respectively. All these angles were less than ideal. In particular, the insertion angle was 29.2° at C6, which was significantly lower than that of the other vertebrae. A possible explanation is that attempting to tilt the screw to 50° may lead to a fracture of the cortical bone of the lamina. Therefore, the insertion angles were adjusted to be slightly lower than the ideal 50°. At C6, the subcutaneous tissue of the posterior neck prevented the screw from tilting, as previously mentioned. However, the screw length exceeded 20 mm in all cases. The typical screw length for LMS was 14-15 mm for the Roy-Camille method and 15-16 mm for the Magerl method [6,7]. The insertion torque should be increased by 4.4 cNm for each 1 mm increase in screw length [5]. Therefore, the LLMS technique can be performed using screws that are at least 5 mm longer than those used for the LMS technique, achieving stronger fixation even at a lower insertion angle of 30°.

The distance between the screw heads for C3-C6 was 13.8 mm, 15.9 mm, 17.6 mm, and 20.4 mm, respectively. The transverse diameters of the spinal cord in healthy individuals reported by Frostell et al. were 12 ± 2.3 mm, 12.8 ± 2.4 mm, 13.3 ± 2.2 mm, and 13.1 ± 1.9 mm for C3, C4, C5, and C6, respectively [8]. The width of the spinal cord should be measured preoperatively and decompressed to 2 mm narrower than the width of the spinal cord to prevent C5 palsy and insufficient decompression [9]. Therefore, considering the reports of various authors, the combination of LLMS and decompression did not result in insufficient decompression.

Laminectomy was performed before posterior decompression for cervical spinal cord compression lesions. In 1978, Hirabayashi et al. reported single-breakout spinal canal enlargement, and in 1982, Kurokawa et al. reported longitudinal split cervical vertebroplasty for cervical vertebral compression lesions [10]. According to studies comparing postoperative neurological symptoms between cervical laminoplasty and cervical laminectomy, clinical outcomes did not differ significantly between the two groups [11,12]. In contrast, long-term results have shown that cervical laminectomy results in postoperative kyphotic deformity [13,14]. Because implant placement is more medial in LLMS than in LMS, cervical laminectomy is the recommended decompression method. However, the neurological prognosis between laminoplasty and laminectomy shows no considerable differences, with minimal risk of kyphosis deformity because of the fusion [12].

Postoperative C5 palsy occurred in one patient (4.5%) in our hospital, which is consistent with those reported in other institutions (2.3-4.6%) [15,16]. Notably, C5 palsy was caused by a complication after decompression and not by nerve root injury due to screw deviation. No cases of neurovascular injury or postoperative dislocation were observed.

There are some limitations to this study. Firstly, it was not examined whether there was a correlation between insertion torque values and screw length. Secondly, it was not examined whether there was any loosening of the screw. Lastly, there was no evaluation of postoperative decompression width. 　

## Conclusions

We reported on LLMS, which has the following problems: the screw angle is inadequate making it difficult to insert a long screw, there is a possibility of inadequate decompression, and cervical laminoplasty cannot be used in combination with LLMS. However, none of these proved to be major problems. LLMS is a safe technique with few complications. In addition, LLMS allows insertion of longer screws than LMS.
